# The HPV Induced Cancer Resource (THInCR): a Suite of Tools for Investigating HPV-Dependent Human Carcinogenesis

**DOI:** 10.1128/msphere.00317-22

**Published:** 2022-08-11

**Authors:** Mikhail Salnikov, Steven F. Gameiro, Peter Y. F. Zeng, John W. Barrett, Anthony C. Nichols, Joe S. Mymryk

**Affiliations:** a Department of Microbiology and Immunology, The University of Western Ontario, London, Ontario, Canada; b Department of Pathology and Laboratory Medicine, The University of Western Ontario, London, Ontario, Canada; c Department of Otolaryngology, The University of Western Ontario, London, Ontario, Canada; d Department of Oncology, The University of Western Ontario, London, Ontario, Canada; e London Regional Cancer Program, Lawson Health Research Institute, London, Ontario, Canada; Northwestern University

**Keywords:** human papillomavirus, cervical cancer, head and neck cancer, TCGA, gene expression, survival, methylation, correlation, oncogene, DNA methylation, analysis resource, cancer, database, HPV

## Abstract

Human papillomaviruses (HPVs) are highly infectious and cause the most common sexually transmitted viral infections. They induce hyperproliferation of squamous epithelial tissue, often forming warts. Virally encoded proteins reprogram gene expression and cell growth to create an optimal environment for viral replication. In addition to their normal roles in infection, functional alterations induced by viral proteins establish conditions that frequently contribute to human carcinogenesis. In fact, ~5% of human cancers are caused by HPVs, with virtually all cervical squamous cell carcinomas (CESC) and an increasing number of head and neck squamous cell carcinomas (HNSC) attributed to HPV infection. The Cancer Genome Atlas (TCGA) molecularly characterized thousands of primary human cancer samples in many cancer types, including CESC and HNSC, and created a comprehensive atlas of genomic, epigenomic, and transcriptomic data. This publicly available genome-wide information provides an unprecedented opportunity to expand the knowledge of the role that HPV plays in human carcinogenesis. While many tools exist to mine these data, few, if any, focus on the comparison of HPV-positive cancers with their HPV-negative counterparts or adjacent normal control tissue. We have constructed a suite of web-based tools, The HPV Induced Cancer Resource (THInCR), to utilize TCGA data for research related to HPV-induced CESC and HNSC. These tools allow investigators to gain greater biological and medical insights by exploring the impacts of HPV on cellular gene expression (mRNA and microRNA), altered gene methylation, and associations with patient survival and immune landscape features. These tools are accessible at https://thincr.ca/.

**IMPORTANCE** The suite of analytical tools of THInCR provides the opportunity to investigate the roles that candidate target genes identified in cell lines or other model systems contribute to in actual HPV-dependent human cancers and is based on large-scale TCGA data sets. Expression of target genes, including both mRNA and microRNA, can be correlated with HPV gene expression, epigenetic changes in DNA methylation, patient survival, and numerous immune features, like leukocyte infiltration, interferon gamma response, T cell response, etc. Data from these analyses may immediately provide evidence to validate *in vitro* observations, reveal insights into mechanisms of virus-mediated alterations in cell growth, behavior, gene expression, and innate and adaptive immunity and may help hypothesis generation for further investigations.

## INTRODUCTION

There are currently over 400 known human papillomavirus (HPV) types (1). These viruses, as exemplified by HPV type 16 (HPV16), contain a small, circular, double-stranded DNA genome of about 8 kbp which encodes 8 or 9 genes. There are 30 HPV types that preferentially infect the anogenital mucosa, causing papillomas (warts) (2). These epithelium-specific viruses are highly transmissible, with peak incidence of infection occurring with the onset of sexual activity (3). Indeed, mucosal HPVs are the most common sexually transmitted infection in North America (4). Furthermore, mucosal HPV types are classified as high risk (HR) or low risk based on their oncogenic potential (2, 5). HR HPV-induced lesions frequently initiate carcinogenesis, commonly as a result of damage to the viral genome, leading to random integration into the host genome. This often leads to a constitutively elevated level of expression of the viral E6 and E7 oncogenes (2). Persistent infection by HR HPVs causes virtually all cervical and other anogenital cancers (2). HR HPV is associated with 85 to 90% of cervical cancers (CESC), with HPV16 predominating via its association with ~50% of all CESC (6, 7). Prognosis for patients with HPV-positive (HPV^+^) CESC is better than for their HPV-negative (HPV^−^) counterparts (8, 9), and it is clear that HPV^+^ and HPV^−^ diseases are clinically and pathologically distinct diseases (10, 11). Worldwide, CESC is the 3rd most prevalent cancer in women, with 570,000 new cases in 2018 (https://gco.iarc.fr/). HPV-induced CESC remains a leading cause of cancer death in younger women in economically disadvantaged countries (2).

In addition to anogenital cancers, HR HPV infection of the oral mucosa is an independent etiological agent of head and neck squamous cell carcinoma (HNSC), which is the 6th most common cancer worldwide, with ~890,000 cases recorded per year (https://gco.iarc.fr/) (12). HNSC include malignant squamous lesions arising in the oral cavity, larynx, pharynx, and oropharynx. HPV infection now accounts for ~25% of all HNSC cases; most HPV^+^ HNSC cases are caused by HPV16, and the remainder are caused by other HR types, with all cases expressing the viral E6 and E7 oncogenes (13–15).

Over the last 30 years, the frequency of HPV-induced oral cancers has increased to epidemic proportions, likely due to changes in sexual behaviors (16, 17). In the United States, more than 70% of oropharyngeal cancers are HPV^+^ (18). Indeed, the incidence of oropharyngeal cancer has been increasing steadily since 1973 in the United States, with base-of-tongue and tonsillar cancers increasing by 2.1% and 3.9% per year, respectively, among white men and women ages 20 to 44 years (19). Similarly, this increase in oropharyngeal cancers was nearly 5% per year between 1970 and 2016 in Sweden (Swedish National Board of Health and Welfare, https://www.socialstyrelsen.se/en/). HPV^+^ HNSC often occurs in younger people, nonsmokers, and nondrinkers compared to their HPV^−^ counterparts (20). Although HPV^+^ tumors generally exhibit favorable clinical outcomes (21, 22), ~15% of these patients do not survive their disease (23, 24). In summary, HPV^+^ HNSC is considered a distinct epidemiological, molecular, and clinical entity (25–27) that is caused by a sexually transmitted virus and is increasing at an epidemic rate (15, 28–30).

HPV^+^ cancers constitutively retain expression of viral genes, primarily the E6 and E7 oncogenes (2, 15), but transcripts for other viral genes are detected in some cancers (31, 32). How these HPV proteins reprogram the infected cell in terms of gene expression, immune response, and metabolism relevant to the development and progression of HPV-induced cancer is a highly active area of investigation (33–36). These studies are complicated by the fact that HPV does not replicate in animal systems, necessitating *in vitro* studies using primary human epithelial cells or human cancer cell lines in culture, *in vivo* studies in transgenic animals, or studies of related animal papillomaviruses as surrogate models of carcinogenesis (37–39). Although tremendously valuable insights into HPV infection and oncogenesis have been made in these surrogate systems, the ability to validate these observations in primary human cancers remains the gold standard. Such validation is often hampered by the lack of human tissue, clinical data, and power to generate statistically robust insights.

The Cancer Genome Atlas (TCGA) represents a large-scale effort by the National Cancer Institute and the National Human Genome Research Institute to create a comprehensive atlas of genomic, epigenomic, and transcriptomic data from primary, surgically resected human cancers (https://www.cancer.gov/tcga). This effort has generated publicly available, comprehensive, multidimensional maps of genomic changes in more than 11,000 tumor samples from 33 distinct types of human tumors. All TCGA samples were processed via a uniform data analysis pipeline that included mRNA sequencing, microRNA (miRNA) sequencing, and DNA methylation profiling. Substantial clinical data are available for many of the TCGA samples, which allows the comparison of many clinical variables, including patient outcome assessment (40).

In terms of HPV-induced cancers, the TCGA CESC data sets are derived from nearly 300 cancer patients (41, 42), while the TCGA HNSC data set is derived from over 500 cancer patients (25, 42). All samples were surgically obtained from treatment-naive individuals, thus avoiding the potentially confounding effects of chemotherapy or radiotherapy on these molecular studies. Initial annotations of tumor HPV positivity and the HPV type present have been expanded and reported in ancillary publications (43, 44), providing the opportunity to make direct comparisons between HPV^+^ tumors and their corresponding HPV^−^ counterparts for many molecular and genetic features. Additionally, viral mRNA read counts for those samples that are positive for HPV16, -33, and -35 (HPV16/33/35^+^) are available (32), as is a systematic analysis of the clinical follow-up intervals for patient survival (40) and estimates of numerous specific immune landscape markers (45). Despite the plethora of useful data in the TCGA and many tools to access it programmatically (46), it remains relatively difficult to analyze this large-scale data primarily by HPV status, particularly for researchers without strong bioinformatic skill sets.

Here we introduce a suite of intuitive web-based tools, The HPV Induced Cancer Resource (THInCR), to assist in querying and visualization of gene expression, methylation, survival, and immune landscape data for both the CESC and HNSC TCGA cohorts. These analysis and visualization tools are intended specifically as a resource for those working in the field of HPV-dependent cancers and should not require any significant computational or bioinformatics expertise. THInCR was developed to allow investigators to rapidly gain greater biological and medical insights by exploring the impact of HPV on cellular gene expression (mRNA and miRNA), altered gene methylation, and associations with patient survival and immune landscape features from primary human cervical and head and neck cancers. The tools can be accessed at https://thincr.ca/, and the stand-alone version can be downloaded at https://github.com/msaland/THInCR-Suite.

## RESULTS

The THInCR suite is a collection of 5 unique tools created for the purpose of exploring a multitude of factors that may be impacted by HPV status in patients with CESC or HNSC. Such factors include differential mRNA and miRNA expression, gene loci methylation, overall patient survival, and changes in immune landscape features. Using the tools present in the THInCR suite, each of these factors can also be correlated with HPV status, genomic location, or gene expression. Analyses are focused on samples positive for the closely related HPV16, -33, and -35 types, which represent the most prevalent infections in both the CESC and HNSC cohorts. This was done to reduce potential variability related to intrinsic functional differences between distantly related HPVs. Table 1 lists the number of samples used in the calculations for each THInCR tool. Note that the CESC cohort has relatively few HPV^−^ or normal control samples, which impacts the ability to define statistically significant differences compared to the more balanced HNSC cohort.

**TABLE 1:** Number of patient samples analyzed from the TCGA CESC and HNSC cohorts for each THInCR tool

Cohort	Patient subset	No. of patient samples analyzed with TCGA tool for:						
		mRNA-seq	miRNA-seq	mRNA vs viral mRNA	miRNA vs viral mRNA	Methylation	Immune comparisons	Survival
CESC	HPV16/33/35^+^	180	180	91	91	180	176	180
	HPV^−^	19	19	NA^*a*^	NA	19	18	19
	Normal control	3	3	NA	NA	3	NA	NA
HNSC	HPV16/33/35^+^	72	64	65	65	72	71	72
	HPV^−^	442	409	NA	NA	442	437	442
	Normal control	40	40	NA	NA	50	NA	NA

a NA, not applicable.

### Differentially expressed gene analysis.

As obligate intracellular parasites, HPVs dramatically reprogram gene expression in the infected cell to provide a more conducive state for the virus replicative cycle (47). As HPV^+^ CESC and HNSC consistently retain expression of a subset of viral genes, including the critical E6 and E7 oncogenes, at least some of these changes in cellular gene expression will likely persist in these cancers.

**FIG 1:**
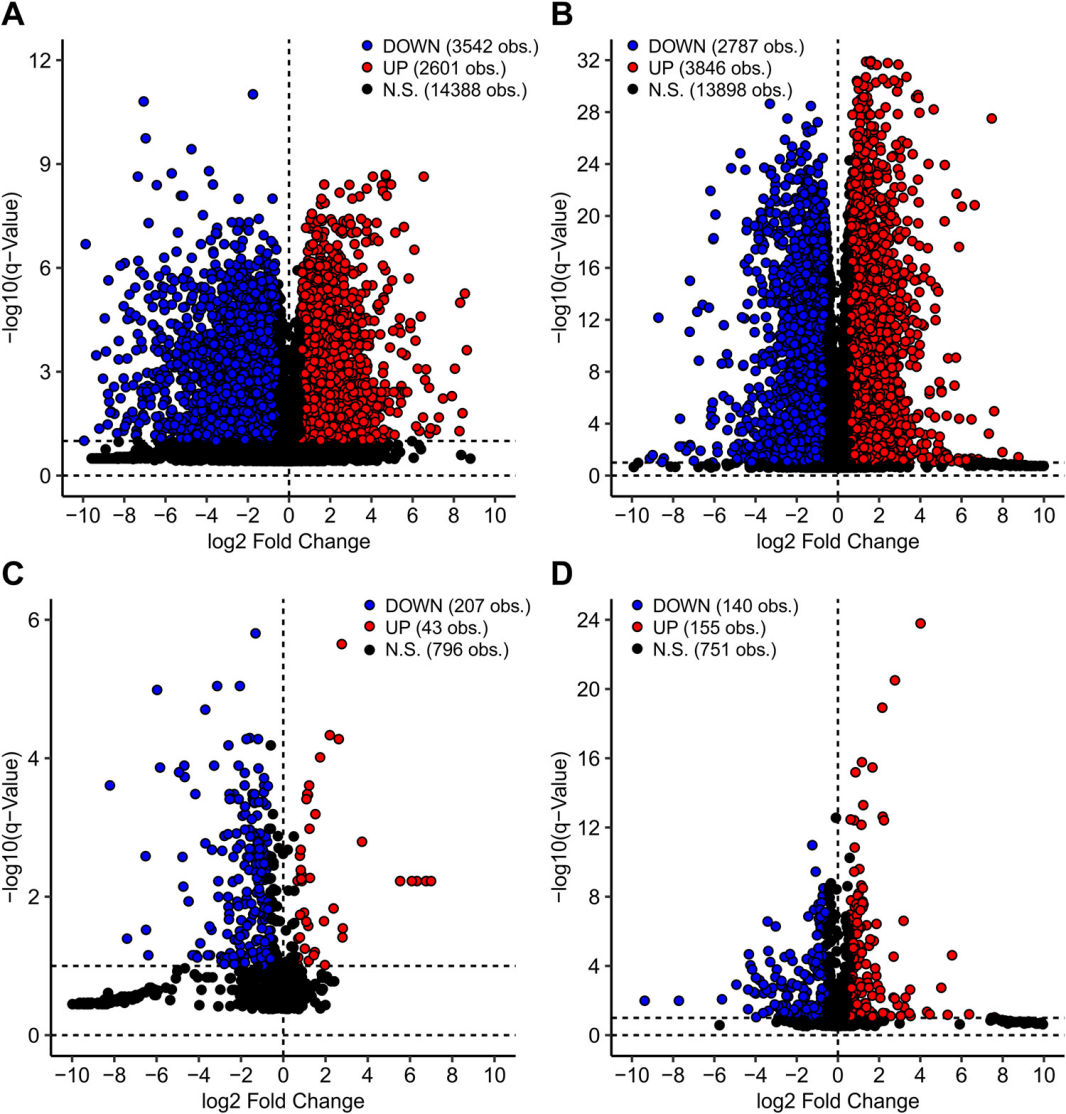
Volcano plots of differentially expressed genes (DEGs) between HPV^+^ and HPV^−^ patients for CESC mRNA (A), HNSC mRNA (B), CESC miRNA (C), and HNSC miRNA (D) TCGA data sets. Each dot represents an individual gene. Genes shaded in blue exhibited a statistically decreased level of expression in HPV16/33/35^+^ cancers. Genes shaded in red exhibited a statistically increased level of expression in HPV^+^ cancers, whereas expression of those indicated in black was not significantly different. Calculations were performed with a false discovery rate (FDR) of 10%.

Thousands of cellular mRNAs and miRNAs are differentially expressed between HPV^+^ cancers and their HPV^−^ counterparts, many of which may be important in the process of carcinogenesis, the maintenance of the cancer phenotype, immune evasion, treatment response, etc. (Fig. 1). The first tool in the THInCR suite is the differential gene expression analysis tool, which allows a user to select a gene of interest (GOI) to determine if it is differentially expressed between HPV^+^, HPV^−^, or normal control tissue, providing easily digestible summaries of this information. This tool can be used to determine if a GOI identified as differentially regulated by HPV in experimental systems is similarly dysregulated in actual primary human cancers from different anatomical sites. Such a validation could indicate that the GOI is worth further pursuit via additional experimentation, allowing the rapid exploration of expression of related genes, and may promote hypothesis generation. Examples of exactly this type of confirmation are already present in the literature (48, 49), but with THInCR these analyses can be performed by anyone in the field. The tool in question is subdivided into two different tools: one for miRNA and the other for mRNA expression patterns, each featuring the CESC and HNSC data sets. These data are restricted to only the HPV16^+^ and closely related HPV33/35^+^ samples from the TCGA CESC and HNSC cohorts.

For each tool, users have the option of selecting a GOI, upon which two different boxplots are generated, one for each featured data set, showing the distribution of expression levels of the selected gene for HPV^+^, HPV^−^, and normal control tissue. Pairwise *P* values and associated *q* values are also calculated, with significant differences highlighted with bright colors, drawing the viewers' attention to statistically significant changes. A table containing statistical information, such as minimum, maximum, and quartile values, for each of the subsets within the boxplots is also generated. The boxplots can be downloaded as .png files, whereas the table with the requisite information to redraw the boxplots can be downloaded as a .csv file, allowing for local data storage for future reference and figure creation. Downloadable tables of all differentially expressed cellular mRNAs and miRNAs are also available through a link provided on the tool’s webpage, to assist users in identifying differentially expressed genes (DEGs) that might merit future investigation.

### Correlation of cellular gene expression with HPV genes.

**FIG 2:**
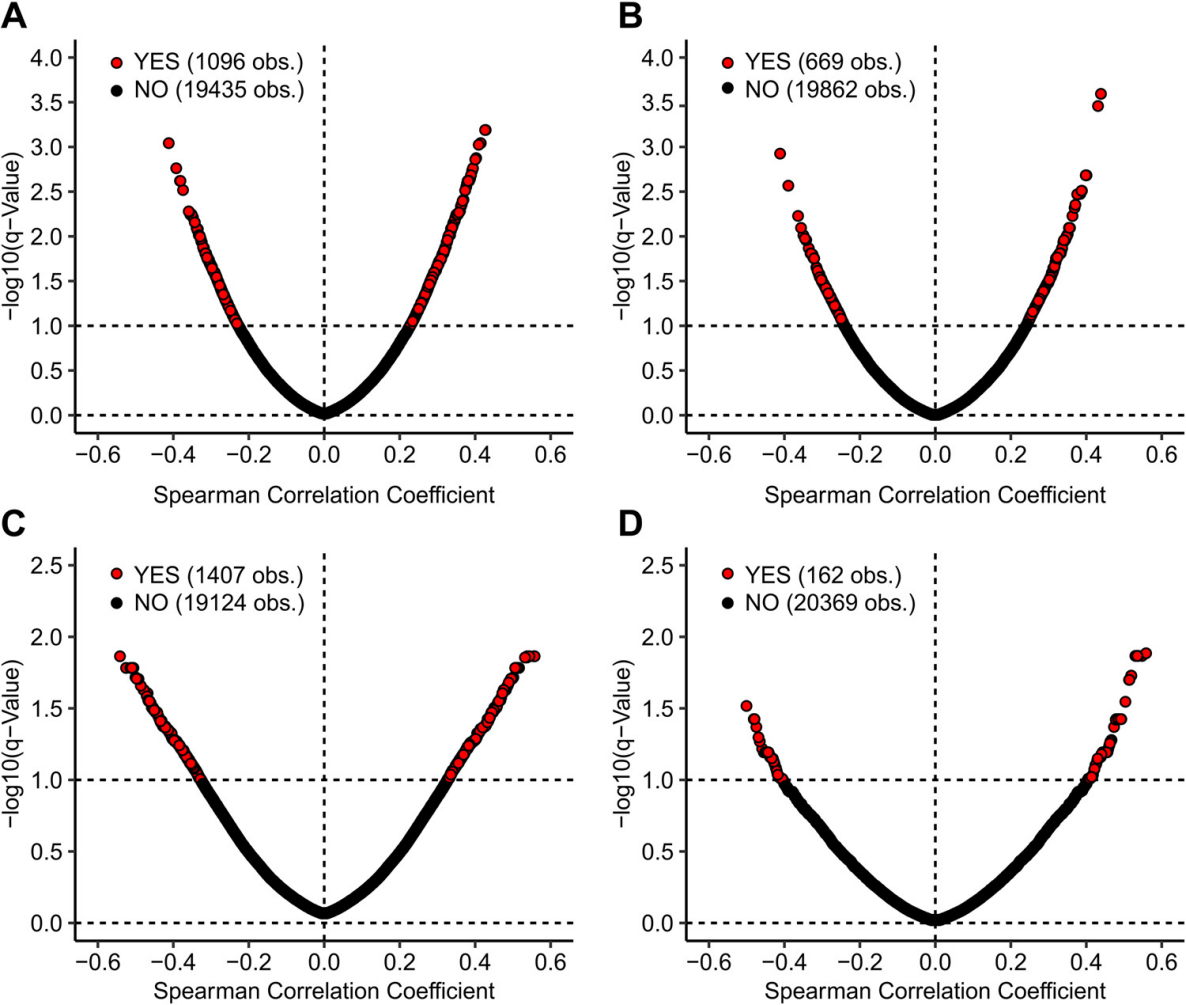
Spearman correlation coefficient versus negative log of significance for CESC cellular mRNA versus E6 (A) or E7 (B) mRNA levels and for HNSC cellular mRNA versus E6 (C) or E7 (D) mRNA. Only HPV16/33/35^+^ samples were included in these analyses. Genes shaded in red exhibited a statistically increased level of expression in HPV^+^ cancers, whereas expression of those indicated in black was not significantly different. Calculations were performed with an FDR of 10%.

The presence of genes that are differentially expressed between HPV^+^ and HPV^−^ cancers suggests that they could be directly or indirectly related to the expression of viral factors retained in these cancers. Furthermore, a causal relationship between a viral gene and altered expression of a cellular gene might be reflected in a proportional or inversely proportional relationship between their expression levels. To investigate this, we utilized the mRNA expression data for each viral gene (E1, E2, E4, E5, E6, E7, L1, and L2) previously reported (32). These data are restricted to only the HPV16^+^ and closely related HPV33/35^+^ samples from the TCGA CESC and HNSC cohorts. The expression of many cellular mRNAs in both CESC and HNSC are significantly correlated with HPV E6 or E7 expression (Fig. 2). The presence of these correlations suggests a causal relationship, which can be investigated as a mechanism or used in hypothesis generation.

The tool in question is subdivided into two different tools: one for miRNA and the other for mRNA expression patterns, each featuring the CESC and HNSC data sets. For each tool, users have the option of selecting a GOI, upon which two different heatmaps are generated, one for each featured data set, showing the correlation between the 8 HPV16/33/35 genes and the selected GOI, with the Spearman correlation score and significance shown for each set of correlations. Correlation scores and pairwise *P* values and associated *q* values are also shown in a separate table, and significant values are highlighted with bright colors, drawing the viewers' attention to statistically significant correlations. The heatmaps can be downloaded as .png files, whereas the table with the heatmap *P* values and *q* values can be downloaded as a .csv file, allowing for local data storage for future reference and figure creation. Downloadable tables of all correlations are also available through the tool’s webpage, via a provided link to assist users in identifying highly correlated genes that might merit future investigation.

### Analysis of the impact of HPV status on gene methylation.

In addition to targeting specific transcriptional regulators like p53 and Rb, HPV-encoded proteins can alter transcription in infected or virally transformed cells via epigenetic mechanisms (33, 50). In particular, genome-wide changes in DNA methylation are clearly observed in both HPV^+^ CESC and HNSC (51, 52). Genome-wide methylation data obtained using the Infinium HumanMethylation450 BeadChip array are available for both the TCGA CESC and HNSC cohorts, allowing the interrogation of the methylation status of ~450,000 CpG sites located throughout the genome (53). Using these data, comparison of the methylation status of HPV^+^, HPV^−^, and normal control tissues revealed a strong HPV-dependent dysregulation of methylation at the *CDKN2A* gene, which encodes p16(INK4A), in CESC and HNSC (54). Thus, these data can be used to provide some insight into the mechanism by which HPV infection induces p16 expression, a surrogate marker historically used for clinical HPV status (55, 56).

**FIG 3:**
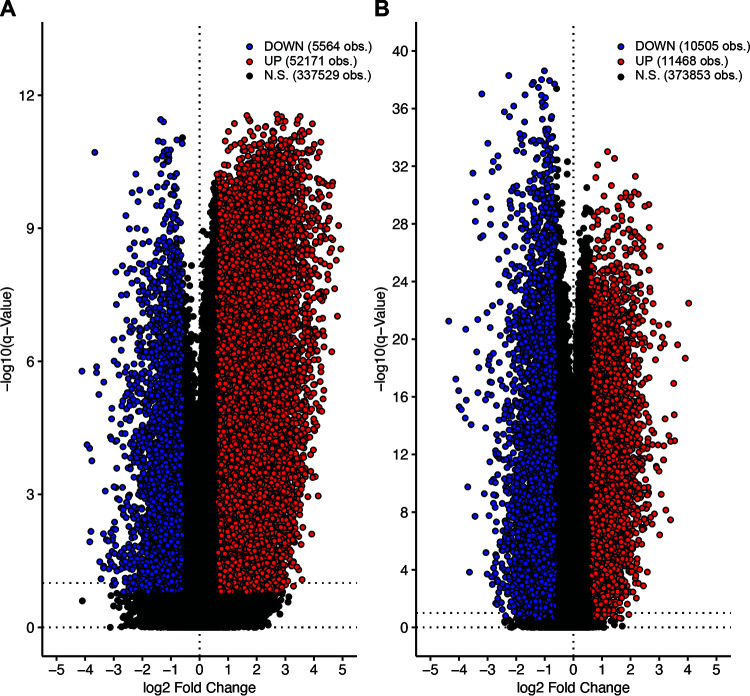
Volcano plots of differentially methylated sites between HPV^+^ and HPV^−^ patients for the CESC (A) and HNSC (B) TCGA data sets. Each dot represents an individual methylation probe from the Infinium HumanMethylation450 BeadChip array. Probes shaded in blue exhibited a statistically decreased level of expression in HPV16/33/35^+^ cancers. Probes shaded in red exhibited a statistically increased level of expression in HPV^+^ cancers, whereas expression of those indicated in black was not significantly different. Calculations were performed with an FDR of 10%.

Both the TCGA CESC and HNSC cohorts exhibited many genomic probes that are differentially methylated between HPV^+^ cancers and their HPV^−^ counterparts (Fig. 3). Many of these methylation sites may be important in the process of carcinogenesis, the maintenance of the cancer phenotype, immune evasion, treatment response, etc. The third tool in the THInCR suite is the differential CpG methylation tool, which allows a user to select a GOI to determine if any probes within it, plus 15% the gene length on either side, are differentially methylated between HPV^+^, HPV^−^, and normal control tissue, providing easily digestible summaries of this information. This tool can be used to determine if a GOI identified as differentially methylated by HPV in experimental systems is similarly dysregulated in actual primary human cancers from different anatomical sites. Such a validation could indicate that the GOI is worth further pursuit via further experimentation, allows the rapid exploration of expression of related genes, and may promote hypothesis generation. Examples of exactly this type of analysis are already present in the literature (49, 54, 57), but with THInCR, this can be performed by anyone in the field. Note that these data are restricted to only the HPV16^+^ and closely related HPV33/35^+^ samples from the TCGA CESC and HNSC cohorts.

This tool encompasses both the miRNA and mRNA expression patterns observed in the CESC and HNSC data sets. For each tool, users have the option of selecting a GOI, along with the genomic region to focus on, which generates line plots for the CESC and HNSC cohorts. These show the average methylation beta value for each genomic probe within the selected region, with average methylation values shown for HPV^+^, HPV^−^, and normal control at a specific probe. The line plot also displays the coding strand for the selected GOI, with a left-to-right arrow representing a forward or Watson strand, whereas the reverse represents the reverse or Crick orientation. A comparison table with *P* values and *q* values is also generated for differences in methylation beta values by HPV status. Significant values are highlighted with bright colors, drawing the viewers' attention to statistically significant differences. Additionally, users can select a probe within the selected region by using a pulldown menu, which generates two boxplots, one for each featured data set. The line plots and boxplots can be downloaded as .png files, whereas the table with the average methylation beta values, *P* values, and *q* values can be downloaded as a .csv file, allowing for local data storage for future reference and figure creation. Downloadable tables of all differentially methylated probes are also available through a link provided on the tool’s webpage, to assist users in identifying areas of methylation that might merit future investigation.

### Impact of gene expression on patient survival.

**FIG 4:**
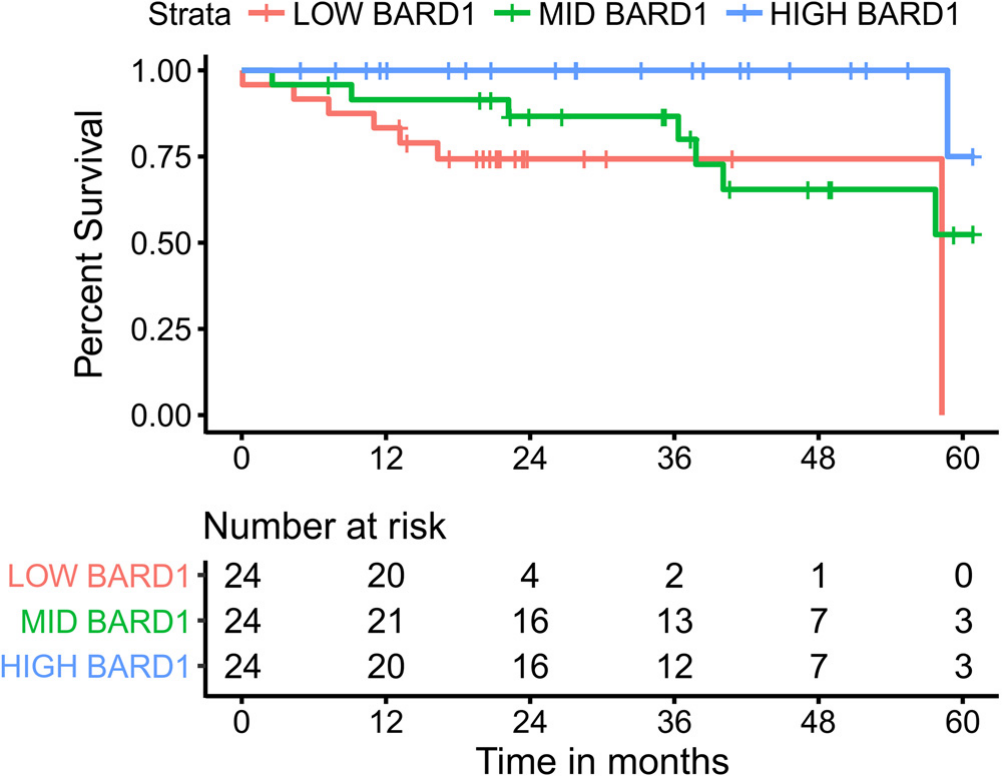
Example survival curve with 3 comparison groups. Analysis was based on mRNA expression levels of BRCA1-associated RING domain 1 mRNA (BARD1; Gene ID 580), with the gene being differentially regulated between HPV^+^ and HPV^−^ samples for HNSC. This figure was generated natively as part of the THInCR suite, as an example of data output. The HPV16/33/35^+^ samples from the TCGA HNSC cohort were divided into high-, middle-, and low-expressing subsets for Kaplan-Meier survival analysis.

High-quality data on patient survival are available for both the CESC and HNSC TCGA cohorts (40), providing the opportunity to determine if altered expression of a GOI is associated with patient outcomes. Many existing studies have exploited these data to identify numerous individual prognostic genes or gene signatures associated with patient survival in the various TCGA, including studies analyzing all 33 cohorts (58). For example, low expression of BARD1 in HNSC shows a significant correlation with patient survival, suggesting that high expression functions in some way as a positive factor for cancer, whether promoting growth, evasion of the immune system, or resistance to genotoxic agents (Fig. 4).

The fourth tool in the THInCR suite allows Kaplan-Meier analysis of patient survival based on expression levels of an individual mRNA or miRNA in both the CESC and HNSC data sets. Once again, these data are restricted to only the HPV16^+^ and closely related HPV33/35^+^ samples from the TCGA CESC and HNSC cohorts. For each tool, users have the option of selecting a GOI, as well as the number of comparison groups, which ranges between 2 and 4. In this way, the cohort is divided into 2, 3, or 4 equally sized subsets based on expression of the target gene for survival comparisons between subsets. Four different survival plots are generated, each representing survival in HPV^+^ CESC, HPV^−^ CESC, HPV^+^ HNSC, and HPV^−^ HNSC. In this way, genes specifically affecting survival in HPV^+^ cancers can be identified, as well as genes associated with outcome independent of HPV status. Pairwise *P* values and associated *q* values are also calculated, with the values shown in a separate region and significant values highlighted with bright colors, drawing the viewers' attention to statistically significant correlations. The survival plots can be downloaded as .png files, allowing for local data storage for future reference and figure creation.

### Impact of gene expression on the immune landscape.

The field of tumor immunology has exploded in the last decade, highlighting the critical role that the immune system plays in tumor development and patient outcomes. The tumor immune landscape is complex, and it has become increasingly clear that it varies widely between different cancer types and even individual tumors of the same type, leading to vastly different treatment responses and patient outcomes (59). Importantly, HPV^+^ HNSC are immune “hot” tumors, with markedly more immune infiltration and higher levels of CD8^+^ T-cell activation than HPV^−^ HNSC (60, 61). Although molecular comparisons of the immune landscape between HPV^+^ and HPV^−^ CESC are scarce, these are pathologically distinct tumors with different clinical outcomes, suggesting that they will also exhibit immunological differences (10, 11). As a “stealth” virus, HPV encodes many distinct immunomodulatory functions that help HPV^+^ tumors evade adaptive immunity (62). Thus, detailed comparisons of the immune landscape between HPV^+^ and HPV^−^ cancers provide an opportunity to identify immunological determinants and the underlying mechanisms by which they are achieved, which may translate to improved treatment of HPV^+^ CESC and HNSC.

**FIG 5:**
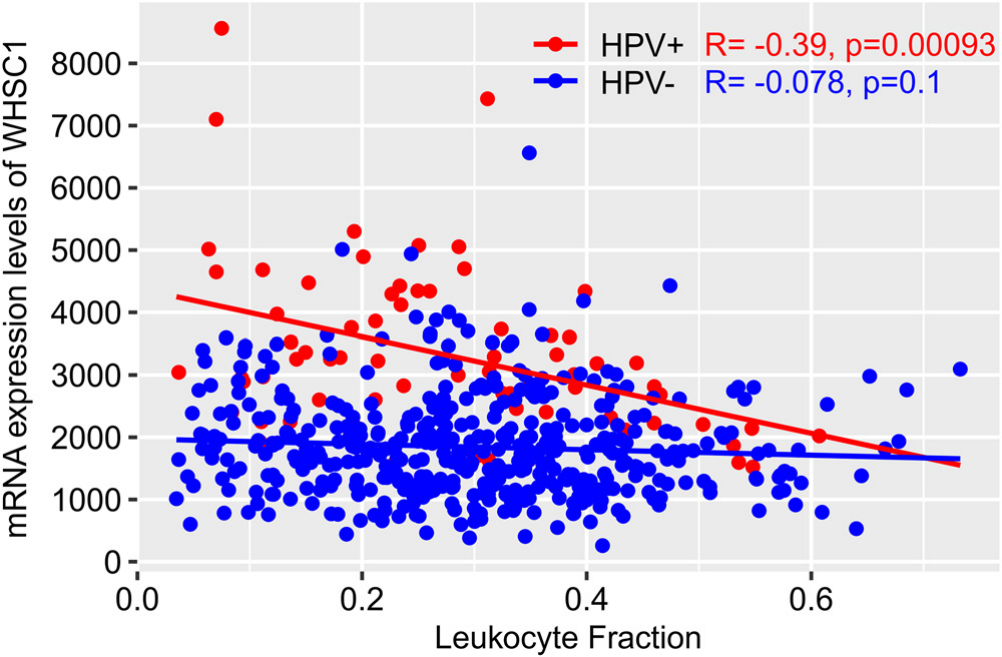
Example of a correlation plot between NSD2/WHSC1 mRNA expression levels and the leukocyte fraction immune landscape feature for the HNSC data set. The figure was generated natively as part of the THInCR suite. Red dots represent HPV16/33/35^+^ HNSC samples, while blue dots represent HPV^−^ HNSC samples. For HPV^+^, *R* = −0.39, *P* = 9.3e−4; for HPV^−^, *R* = −0.078, *P* = 0.1.

A recent study entailed an extensive immunogenomic analysis of more than 10,000 tumors from the TCGA (45). This analysis was used to construct the final tool in the THInCR suite, which allows a user to investigate the impact of expression of a GOI, either mRNA or miRNA, on numerous immune landscape features (Table 2). These analyses are restricted to only the HPV16^+^ and closely related HPV33/35^+^ samples from the TCGA CESC and HNSC cohorts. Users have the option of selecting a GOI, upon which a table featuring 53 immune landscape features is generated, showing correlation and significance for each of the 4 categories (CESC HPV^+^, CESC HPV^−^, HNSC HPV^+^, HNSC HPV^−^), with significant values highlighted with bright colors, drawing the viewers' attention to statistically significant correlations. Users then have the option of selecting an immune landscape feature, upon which two scatterplots and two boxplots are generated, one for each featured data set (see the example analysis in Fig. 5). The line plots and boxplots can be downloaded as .png files, whereas the table with the correlation and significance scores can be downloaded as a .csv file, allowing for local data storage for future reference and figure creation.

**TABLE 2:** The 53 immune landscape features available for analysis in the THInCR suite of tools

Immune landscape feature^*a*^	
Aneuploidy score	Monocytes
B cells memory	Neutrophils
B cells naive	NK cells activated
BCR evenness	NK cells resting
BCR richness	Nonsilent mutation rate
BCR Shannon	No. of segments
CTA score	Plasma cells
Dendritic cells	Proliferation
Dendritic cells activated	Silent mutation rate
Dendritic cells resting	SNV neoantigens
Eosinophils	Stromal fraction
Fraction altered	T cells CD4 memory activated
Homologous recombination defects	T cells CD4 memory resting
IFN gamma response	T cells CD4 Naive
Indel neoantigens	T cells CD8
Intratumor heterogeneity	T cells follicular helper
Leukocyte fraction	T cells gamma delta
Lymphocyte infiltration signature score	T cells regulatory Tregs
Lymphocytes	TCR evenness
Macrophage regulation	TCR richness
Macrophages	TCR Shannon
Macrophages M0	TGF beta response
Macrophages M1	Th1 cells
Macrophages M2	Th17 cells
Mast cells	Th2 cells
Mast cells activated	Wound healing
Mast cells resting	

a BCR, B cell receptor; CTA, cancer testis antigens; IFN, interferon; SNV, single nucleotide variant; TCR, T cell receptor; TGF, transforming growth factor.

## DISCUSSION

High-risk HPV infection continues to be responsible for ~5% of human cancers, despite the availability of subunit vaccines against HPV since 2006. These vaccines induce neutralizing antibodies that are predominantly type specific (63). Importantly, these vaccines are prophylactic, rather than therapeutic, as they target the L1 virion protein, which is not often expressed in HPV^+^ cancers. While vaccination will reduce HPV-induced cancer in the long term, there would still be many infections caused by HPV types not neutralized by these vaccines, which include the nonavalent vaccine (64). In addition, these vaccines do not benefit the millions of people with existing HPV infections and are prohibitively expensive in many areas with high HPV infection rates. Thus, despite excellent vaccines, HPV will likely remain an important human pathogen, and a major cause of cancer, over the long term (65). As a result, studies aimed at understanding the molecular basis by which HPV reprograms the infected cell, predisposing it to oncogenic transformation, remain an important area of tumor virus research that is directly relevant to human health.

The large-scale multimodal data generated by the TCGA remain a tremendous resource for cancer research, including tumor virus research. Although level 3 preanalyzed high-level data are freely available to any user via the Broad GDAC Firehose (https://gdac.broadinstitute.org/), their utility to wet lab researchers and clinicians with limited computational skills is limited without a significant investment of time and effort. This has spawned a plethora of web-based utilities that can greatly assist most areas of investigation (46, 66). THInCR was created specifically with the needs of the HPV research community in mind and using the CESC and HNSC TCGA cohorts. It provides access to a gene-centered approach to study the impact of HPV status on cellular mRNA and miRNA gene expression and DNA methylation. Furthermore, it allows the user to explore correlations with expression of individual HPV genes, patient survival, and immune landscape features. While there are notable advantages to using these large-scale data, an important limitation is that these tools are almost entirely based on RNA expression levels, which may not accurately reflect actual protein expression and/or activity, as described in detail by others (45). Importantly, THInCR uses actual molecular annotation to define true HPV status, rather than p16 status as a surrogate marker, as originally reported for the TCGA HNSC cohort (26). The simple, intuitive, and interactive interface facilitates the visualization, interpretation, and acquisition of otherwise-complex information relevant to studies of HPV oncogenesis that are not reachable in other packages. Basic and clinical researchers working in this field can readily explore the features of their favorite gene in two different anatomical sites, while comparing expression, methylation, survival, and immune landscape changes between HPV^+^ and HPV^−^ disease.

Another important feature provided by THInCR is the ability to download graphical interpretations of each selected analysis suitable for documenting the analysis or presenting it informally. Importantly, at the press of a button, sets of tabular data in a format easily amenable for advanced customized graphical rendering for publication can also be downloaded. Additionally, master files of DEGs, differentially methylated probes, and gene expression correlations with viral genes can also be downloaded for further analysis or reference by the user.

In conclusion, THInCR is an intuitive interface to explore and interpret HPV-dependent changes in gene expression and DNA methylation using molecularly annotated CESC and HNSC data sets from the TCGA. HPV^+^ versus HPV^−^ versus normal control tissue comparisons are possible in the form of graphs and comprehensive tables. These data can provide evidence to validate the tumor relevance of existing experimental observations in model systems and/or facilitate novel hypothesis generation. THInCR is freely accessible at https://thincr.ca/.

## MATERIALS AND METHODS

### Implementation and software.

The web server has been deployed on Amazon Web Services (AWS) running Ubuntu version 22.04, Shiny Server version 1.5.18.987, and R version 4.2.0. The following packages, along with required dependencies, have been installed: Shiny version 1.7.1, dplyr version 1.0.9, shinythemes version 1.2.0, data.table version 1.14.2, ggplot2 version 3.3.6, gdata version 2.18.0.1, reshape2 version 1.4.4, ggpubr version 0.4.0, DT version 0.23, plotly version 4.10.0, tidyverse version 1.3.1, survival version 3.3-1, survminer 0.4.9, Hmisc version 4.7-0, and scales version 1.2.0. Server setup was performed according to Bordet’s instructions at https://www.charlesbordet.com/en/guide-shiny-aws/. The service is available 24 h/day, 7 days/week at https://thincr.ca/, barring downtime for maintenance. Tutorial videos for tools are available through the home page under the section “Video Guides.” A stand-alone version of THInCR for Windows can be downloaded at https://github.com/msaland/THInCR-Suite.

### Sample collection and ethics.

All data were downloaded from the Cancer Genome Atlas (TCGA) via the Broad Genome Data Analysis Center’s Firehose server (https://gdac.broadinstitute.org/) or other publicly available sources as noted below; therefore, no ethical approval was needed. Table 1 lists the number of available samples used in the calculations for each THInCR tool.

### Data sources for mRNA and miRNA expression levels, patient cohort composition, and analysis workflow.

Level 3 mRNA and miRNA expression data for the TCGA HNSC and CESC data sets were sourced from Broad Genome Data Analysis Center’s Firehose server (https://gdac.broadinstitute.org/), with the data sets manually annotated as described by Gameiro et al. (67). The mRNA data sets feature expression patterns of 20,533 unique genes, whereas the miRNA data sets feature 1,048 unique genes. The CESC mRNA and miRNA data sets are each comprised of 165 HPV16^+^, 9 HPV33^+^, and 6 HPV35^+^ samples. The HNSC mRNA data set is comprised of 61 HPV16^+^, 8 HPV33^+^, and 3 HPV35^+^ samples. The miRNA data set is comprised of 54 HPV16^+^, 7 HPV33^+^, and 3 HPV35^+^ samples. Boxplots were generated using the ggplot2 package (version 3.3.5). Maximum and minimum boxplot values are represented as 1.5× upper and lower quartile ranges, respectively. The correlation of cellular gene mRNA or miRNA expression and HPV status was performed via sorting the data set into HPV16/33/35^+^, HPV^−^, or normal (noncancerous) subsets, with subsequent calculations performed with R’s built-in wilcox.test function with the conf.level parameter set to 0.95. Patient cellular gene mRNA and miRNA expression with >50% zero or null values were marked as nonsignificant regardless of calculated *P* value. *q* values were calculated for each comparison group with a false-discovery rate (FDR) of 10%.

### Data sources for viral mRNA expression levels, patient cohort composition, and analysis workflow.

The HPV16/33/35 viral mRNA expression data sets were sourced from Ren et al. (32), with the data sets manually annotated as described by Gameiro et al. (67). These data sets feature expression levels of the E1, E2, E4, E5, E6, E7, L1, and L2 genes expressed by a subset of HPV16^+^, HPV33^+^, and HPV35^+^ TCGA CESC and HNSC patients, with expression being summed into a single value for individual genes with multiple mRNAs. The CESC data set features 91 patient observations, with 88 HPV16^+^, 2 HPV33^+^, and 1 HPV35^+^ sample. The HNSC data set features 65 patient observations, with 54 HPV16^+^, 8 HPV33^+^, and 3 HPV35^+^ samples. The correlation of HPV16/33/35 mRNA and cellular gene mRNA or miRNA expression was performed via R’s built-in cor.test function, with the function being run with the linear relationship and Spearman correlation coefficient arguments. Patient cellular gene mRNA or miRNA and HPV mRNA expression with >50% zero or null values were marked as nonsignificant regardless of calculated *P* value. *q* values were calculated for each comparison group with an FDR of 10%. Boxplots and scatterplots were generated using the ggplot2 package (version 3.3.5). Maximum and minimum boxplot values are represented as 1.5× upper and lower quartile ranges, respectively.

### Data sources for DNA methylation levels, patient cohort composition, and analysis workflow.

Level 3 Infinium HumanMethylation450 BeadChip array data sets for the TCGA HNSC and CESC cohorts were sourced from the Broad Genome Data Analysis Centers Firehose server (https://gdac.broadinstitute.org/), with the data sets manually annotated for HPV status as described by Gameiro et al. (67). The data sets feature the methylation beta values for 395,329 different probes, along with chromosome number and genomic coordinates. The CESC data set features 303 patient observations, with 180 HPV16/33/35^+^, 19 HPV^−^, and 3 normal controls. The HNSC data set features 558 patient observations, with 72 HPV16/33/35^+^, 442 HPV^−^, and 50 normal controls. The correlation of probe methylation beta value and genomic loci was performed via sorting the data set into HPV16/33/35^+^ and HPV^−^ subsets, with subsequent calculations performed via R’s built-in wilcox.test function with the conf.level parameter set to 0.95. Probe methylation beta with >50% zero or null values were marked as nonsignificant regardless of calculated *P* values. *q* values were calculated for each comparison group with an FDR of 10%. Boxplots and line plots were generated using the ggplot2 package (version 3.3.5). Maximum and minimum boxplot values are represented with 1.5× upper and lower quartile ranges, respectively.

### Data sources for patient survival, patient cohort composition, and analysis workflow.

The TCGA HNSC and CESC overall survival (OS) data sets were sourced from Liu et al. (40), with the data sets manually annotated for HPV status as described by Gameiro et al. (67). The CESC data set has 180 HPV16/33/35^+^ and 19 HPV^−^ patient observations. The HNSC data set has 72 HPV16/33/35^+^ and 442 HPV^−^ patient observations. The correlation of survival and cellular gene mRNA and miRNA expression was performed via sorting the data set into HPV16/33/35^+^ and HPV^−^ subsets, with subsequent calculations performed via the pairwise_survdiff and Surv functions, available via survminer and survival packages, respectively. Users have the option of selecting the number of comparison groups, upon which the subsets are broken down by the selected quantile. Patient cellular gene mRNA and miRNA expression with >50% zero or null values were marked as nonsignificant regardless of calculated *P* values. *q* values were calculated for each comparison group with an FDR of 10%. Kaplan-Meier survival plots were generated using the ggsurvplot function available through the survminer package (version 0.4.9).

### Data sources for immune landscape features, patient cohort composition, and analysis workflow.

The immune landscape features for the TCGA HNSC and CESC data sets were sourced from Thorsson et al. (45), with the data sets manually annotated for HPV status as described by Gameiro et al. (67). The data sets included 53 immune landscape features as listed in Table 2. The correlation of immune landscape features and cellular gene mRNA and miRNA expression was performed via sorting the data set into HPV^+^ and HPV^−^ subsets, with subsequent calculations performed via R’s built-in cor.test function, with the function being run with the linear relationship and Spearman correlation coefficient arguments. Immune landscape features and patient cellular gene mRNA and miRNA expression with >50% zero or null values were marked as nonsignificant regardless of calculated *P* values. *q* values were calculated for each comparison group with an FDR of 10%.
